# West Country Physicians Club

**Published:** 1983-10

**Authors:** 


### West Country Physicians' Club

The Seventy-second meeting of the West Country Physicians' Club was held in Plymouth on Friday, 29th and Saturday, 30th April 1983. The Plymouth physicians acted as hosts.

On Friday cases were shown and a very successful dinner was held at the Moorland Links Hotel.

The following new members were unanimously elected:

Dr. Peter Hickling
Rheumatologist
Plymouth

Dr. David Hamilton
General Physician/Renal Disease
Truro

Dr. Stuart Glover
Infectious Disease
Ham Green Hospital

Dr. Peter Harrison
Nephrologist
Southmead Hospital

Dr. Clive Roberts
Consultant Senior
Lecturer/Clinical
Pharmacologist
Bristol Royal Infirmary

On Saturday morning the following papers were presented (*abstracts appear below):

A singular case of acromegaly
Professor D. Mattingly

* Continuous ambulatory peritoneal dialysis
Dr. H. Leather

* The beneficial effects of moderate alcohol intake on bile and blood
Dr. K. W. Heaton

* Parkinson, politics, palaeontology, pharmacology and Plymouth
Dr. K. R. Hunter

After coffee on Saturday, Dr. David Keeling, Adviser in Nuclear Medicine, Plymouth, addressed the Club.

The Plymouth physicians must be congratulated on an extremely successful meeting. The next meeting of the Club will take place at Bristol Royal Infirmary on Friday, 4th and Saturday, 5th November 1983.

### CONTINUOUS AMBULATORY PERITONEAL DIALYSIS

**H. Leather, Plymouth**

Continuous ambulatory peritoneal dialysis (CAPD) is the most important advance in replacement of renal function in recent years.

Intermittent peritoneal dialysis twice weekly for 24–36 hours has been carried out for many years. CAPD as its name implies is a continuous process. The patient learns the technique over 2 weeks and is then independent. Using the strictest aseptic technique the patient attaches a 2 litre plastic bag of dialysing fluid to the soft rubber peritoneal catheter and runs it into the abdomen. The empty bag is then strapped to the abdomen and 6 hours later the patient drains the fluid from the abdomen by gravity. The bag is then disconnected from the tubing, a new bag attached and the process repeated. This is carried out four times daily.

The advantages of CAPD are an increased sense of well being, reduced fluid and dietary restriction, steady state biochemistry, increased haemoglobin level and easier management of hypertension. The only substantial disadvantage is peritonitis.

In Plymouth CAPD was started in November 1979 but discontinued in June 1981 owing to nursing shortage. In this initial period there were 18 patients and at March 1983, 8 were alive. Of the 10 deaths (average 55 years) one was from peritonitis, the remainder from cerebral and cardio-vascular disease. CAPD was restarted in April 1982 and by March 1983 26 patients had been taken on, of whom 25 were still living. In both groups peritonitis was the most important single problem. Overall results showed that the majority of patients were back at work. Of the 21 patients who had previously had experience of haemo-dialysis, 15 felt subjectively better, 6 however had transferred to haemo-dialysis because of technical failure, persisting infection or abdominal pain.

Overall, CAPD is the dialysis treatment of choice in the Plymouth Unit.

### BENEFICIAL EFFECTS OF MODERATE ALCOHOL INTAKE ON BLOOD AND BILE

**K. W. Heaton and J. R. Thornton, Bristol**

Epidemiologically, there is strong evidence that drinking alcohol reduces the risk of coronary heart disease and raises plasma high-density lipoprotein (HDL) cholesterol. There is weak epidemiological evidence that drinking alcohol may protect against gallstones.

Bile supersaturated with cholesterol is the fundamental pre-requisite for cholesterol gallstone formation. We have previously demonstrated an inverse relation between the cholesterol saturation of bile and plasma HDL cholesterol and suggested that factors which affect one of them might affect the other in the opposite way. Experimentally, alcohol probably raises HDL cholesterol but the data are limited and conflicting. The effect of alcohol on bile is unknown.

To test whether alcohol reduces bile cholesterol saturation, 12 healthy volunteers with a very low initial alcohol intake drank 39 g alcohol daily for 6 weeks, followed by 6 weeks abstention from alcohol. Fasting blood and duodenal bile were sampled on three occasions.

HDL cholesterol (mean±SEM) rose from 1.07±0.05 to 1.25±0.08 mmol/l (41.4±1.9 to 48.3±3.1 mg/dl) with alcohol ($P<0.01$) and fell to 1.04±0.06 mmol/l (40.3±2.3 mg/dl) with abstention ($P<0.001$). Conversely, bile cholesterol saturation index fell from 1.31±0.06 to 1.08±0.06 with alcohol ($P<0.001$) and rose to 1.27±0.09 with abstention ($P<0.01$). Bile saturation index and HDL cholesterol were inversely correlated ($r=-0.56$, $P<0.001$).

The data provide further evidence of a biochemical link between cardiovascular disease and cholesterol gallstones and suggest that moderate alcohol intake has some protective effect against both diseases.

### PARKINSON, POLITICS, PALAEONTOLOGY, PHARMACOLOGY AND PLYMOUTH

K. R. Hunter, Plymouth

Several people, for example Rowntree (1982) and Jefferson (1973) have given good accounts of James Parkinson's life. Some aspects are of particular interest to Physicians in the West Country. He was an active political reformer, urging changes in medical education (Parkinson, 1800) which were later championed by the Devonshire doctor, Thomas Wakley. He wrote about palaeontology and he was aware of the rich fossil beds in South Devon. He had an enlightened attitude to the clinical use of drugs and it is legitimate to regard him as a physician with a special interest in clinical pharmacology (Hunter, 1977).

A complete set of notes, taken by a student at John Hunter's lectures on the Theory and Practice of Surgery in 1781, was recently discovered in Plymouth. James Parkinson also attended these lectures (Parkinson, 1833) and it has intrigued neurologists that Parkinson's only serious error in his Essay on the Shaking Palsy (Parkinson, 1817) was to state that the tremor becomes worse during sleep (Calne, 1970). Comparison of Parkinson's notes about Hunter's Lecture V with the notes discovered in Plymouth suggests that Parkinson was so preoccupied by Hunter's account of dreams that he didn't pay his usual meticulous attention and he missed Hunter's explicit statement that extra pyramidal tremor ceases during sleep.

